# Noise and Electrical Characteristics of Composites Filled with Onion-Like Carbon Nanoparticles

**DOI:** 10.3390/polym13070997

**Published:** 2021-03-24

**Authors:** Marina Tretjak, Edita Palaimiene, Sandra Pralgauskaitė, Jonas Matukas, Jūras Banys, Jan Macutkevič, Vanessa Fierro, Sébastien Schaefer, Alain Celzard

**Affiliations:** 1 Institute of Applied Electrodynamics and Telecommunications, Vilnius University, LT-10257 Vilnius, Lithuania; marina.tertjak@ff.vu.lt (M.T.); edita.palaimiene@ff.vu.lt (E.P.); sandra.pralgauskaite@ff.vu.lt (S.P.); jonas.matukas@ff.vu.lt (J.M.); juras.banys@ff.vu.lt (J.B.); 2 French National Centre for Scientific Research, Institute Jean Lamour, Université de Lorraine, F-88000 Epinal, France; vanessa.fierro@univ-lorraine.fr (V.F.); alain.celzard@univ-lorraine.fr (A.C.); sebastien.schaefer@univ-orleans.fr (S.S.)

**Keywords:** charge carrier transfer, composite, electrical conductivity, fluctuation, noise, onion-like carbon

## Abstract

Polymer matrix composites filled with carbon nanoparticles are promising materials for many applications, but their properties strongly depend on the particle features, concentration and distribution within the matrix. Here we present a study of the electrical resistivity and the low-frequency voltage fluctuation of composites based on epoxy resin filled with onion-like carbon (OLC) of different sizes (40–250 nm) above the percolation threshold, which should clarify the electrical transport characteristics in these materials. Electrical measurements were performed in the temperature range of 78 to 380 K, and voltage noise analysis was carried out from 10 Hz to 20 kHz. At low temperatures (below 250 K), thermally activated tunneling, variable-range hopping and generation–recombination of charge carriers take place. Above 250 K, the rapid expansion of the matrix with the temperature increases the resistivity, but above ~330 K, the conductivity of the matrix becomes significant. Quasi one-dimensional electrical transport is observed in composites with the smallest particles (40 nm), while in composites with the largest particles (220–250 nm), the dimensionality of the electrical transport is higher. The temperature dependence of the electrical conductivity of composites with smaller particles is more sensitive to matrix expansion.

## 1. Introduction

Carbon is a chemical element with a wide variety of structures, leading to a broad range of physical properties. These remarkable characteristics are attracting increasing attention, and have led to numerous studies of carbon-containing materials [1,2,3]. Fullerenes are one of the forms of carbon that have stimulated study of these materials and similar structures, and have practically formed a new branch in themselves in materials science and nanotechnology. It has taken time, but interest in materials based on these structures has not only not weakened, but continues to grow [4,5,6]. The large multi-shelled carbon fullerenes are called onion-like carbons (OLCs). They always have structural defects and high electrical conductivity, like carbon nanotubes [7]. For this reason, they are often used for various electronic applications, in sensors and for electromagnetic shielding [8].

The development of modern technologies forces scientists to search for new materials with unique properties. Composite materials based on nanoparticles have many useful properties: increased strength and hardness, low weight and a wide range of interesting thermophysical and electrophysical parameters [9]. The unique properties of these composites make them technologically superior, more economical than other materials and more adaptable to a variety of applications [10,11]. Composites based on carbon nanostructures are successfully used in electronics for devices that actively interact with electromagnetic radiation by reflecting or absorbing microwaves [6,12]. Indeed, the proliferation of new electronic equipment operating in the microwave range of the electromagnetic spectrum (such as personal computers, microwave ovens and various (wireless) information exchange devices) requires protection against high-frequency electromagnetic fields, which affect neighboring devices and organisms, including humans [13]. Such materials are also used to reduce interference from radio components, to improve the electromagnetic compatibility of various systems and environmental conditions [14,15]. Effective shielding is also used for commercial and defense equipment [16,17]. On the other hand, composites with carbon nanostructures have a positive temperature coefficient in the temperature range of about 250 K to 325 K, making them suitable as current limiting devices [18]. These materials are also finding applications in the growing Li-ion battery [19,20] and capacitor [5] industries. Onion-like carbon has been shown to have better pseudo-capacitive performance than other carbon nanoparticles [5]. OLC, which is the filler of the composites studied in this paper, is also a good raw material for photonic applications, due to its effective optical limiting effect [21]. Indeed, OLC particles contribute to broad UV adsorption at 217.5 nm, which is also the case in interstellar dust [22]. Composites with OLC are also promising electrode materials for energy storage and conversion, due to their very low electrical resistance [4,23,24]. Finally, the advantage of OLC particles is that their manufacture by annealing nanodiamonds is relatively simple [4].

Whatever the application, a complete understanding of the characteristics of any new composite and the influence of its fillers on the electrical, optical and other properties of the material is essential. To this end, experimental research on composites based on various carbon nanoparticles has been conducted and theoretical models have been developed for several decades [25,26,27]. However, there are still not enough research to completely understand all the properties of composites, which depend on many characteristics of the fillers (type, dimensions and distribution, orientation of the nanoparticles, their concentration, etc.) [2,3,6]. Attempts to theoretically describe the electrical properties have been made since the beginning of research on composites with carbon fillers [25,26,27,28,29,30], but only the experimental study of conductivity mechanisms allows a real understanding of the charge transfer between the conductive filler grains in a polymer matrix. This information is necessary for a complete understanding of the characteristics of the materials and the limitations of devices based on them. Thermal activation of charge carriers, tunneling, hopping, fluctuation-assisted hopping and other carrier transfer mechanisms have already been mentioned elsewhere [12,28,29,30,31], but it is also observed that the dominant mechanism depends on the characteristics and concentration of the conductive filler particles and varies with operating conditions, e.g. temperature. Electrical transport of OLC embedded in polyurethane and polydimethylsiloxane were investigated in a wide temperature range [32,33,34]. It was established that the electrical transport in these composites occurs to due electrons hopping inside OLC clusters and their tunneling between conductive clusters [32,33,34]. However, the electrical noise was not investigated in composites with OLC inclusions up to now.

One of the most sensitive methods for analyzing charge transfer mechanisms in composite materials is the study of low-frequency noise, which allows the smallest fluctuation in the physical processes occurring in the material to be captured [35,36,37,38]. There are very few papers on the noise characteristics of carbon nanoparticle-based composite materials [35,39], although noise studies can highlight the influence of the type of carbon nanofiller on the charge transfer mechanisms [40,41]. In this paper, we present an in-depth study of the electrical and low-frequency noise characteristics of composite materials where onion-like carbon particles of different sizes have been used as conductive fillers in an epoxy resin. This study was carried out with the aim of clarifying the mechanisms of charge carrier transfer in these materials and their dependence on particle size and concentration. It has been shown that thermally activated tunneling, variable-range hopping and charge carrier generation and recombination control the electrical conduction in these materials.

## 2. Materials and Methods

### 2.1. Investigated Composite Materials with Onion-Like Carbon Filler

**Figure 1:**
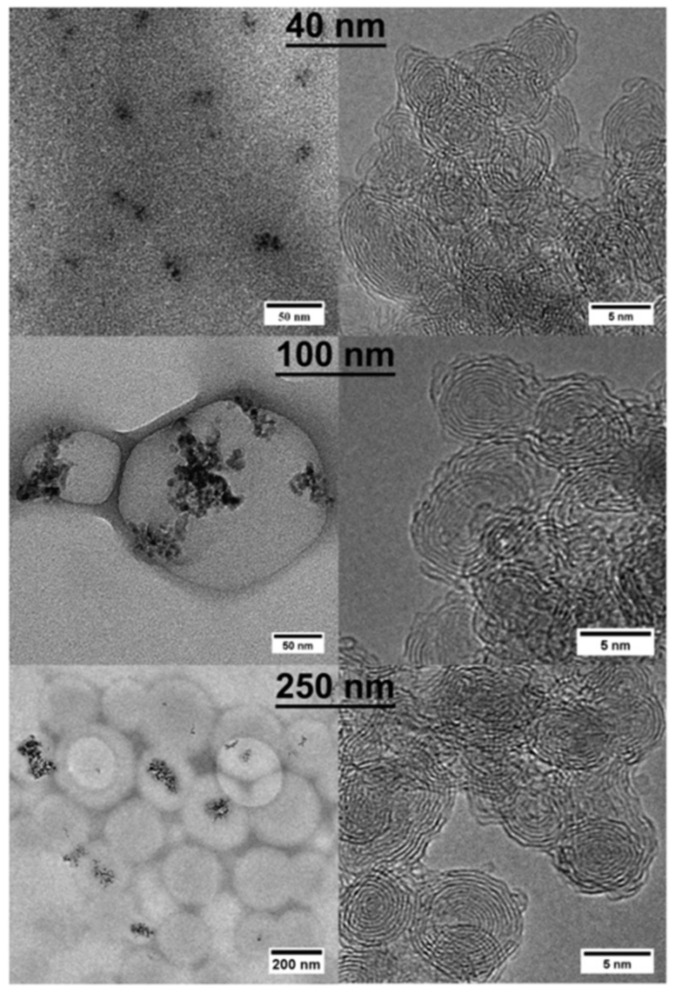
Transmission electron microscopy (TEM) images of onion-like carbon (OLC) aggregates, deposited on a carbon lacey TEM grid, having 3 different average sizes: 40 nm (top), 100 nm (middle) and 250 nm (bottom line). Left and right columns correspond to low and high magnification images, respectively.

The research presented in this article deals with onion-like carbon–epoxy composites. These materials have been shown to have good electrical conductivity, which allows their use in various electrical and electronic circuits [42,43]. The structure of onion-like carbon is a nested fullerene-type sphere, which is combined into units by several defective outer shells [4,6]. The production of OLC is not complex and is cheaper than the production of carbon nanotubes. The OLC particles of the studied composite materials were manufactured from detonation nano diamonds, which consist of aggregates based on primary particles of size 4–6 nm. The ultra-dispersed diamonds were oxidized in concentrated sulfuric acid and chromic anhydride at 383 K. The particles were then washed and dried. Finally, the particles were annealed for 3 hours at 1923 K in vacuum. Detonation nanodiamonds were obtained from the explosion under gaseous carbon dioxide of a mixture of trinitrotoluene and 1,3,5-trinitroperhydro-1,3,5-triazine [7]. The resultant material was centrifuged so that 3 fractions were recovered. This method of manufacturing OLC has been used since 1994 and is described in more detail in [7]. A dynamic light scattering technique proved that the aggregate size distributions of these fractions were narrow and centered on 40, 100 and 250 nm, and that their conversion into OLC did not change these characteristics. These features were checked by suspending OLC and DNDs in N-methyl-2-pyrrolidone and water, respectively, in which the corresponding powders can be well-dispersed. OLC aggregates were also observed by transmission electron microscopy (TEM) using a JEOL JEM 2010 electron microscope (JEOL Ltd, Tokyo, Japan). OLC agglomerates from the three different fractions are shown in Figure 1, which present TEM pictures. There are no individual particles but, the primary particles are clustered. Figure 1 also shows that there is no difference between the primary particles or in the way they are averaged; only the size of agglomerates is different, depending on the fractions obtained after centrifugation.

**Figure 2:**
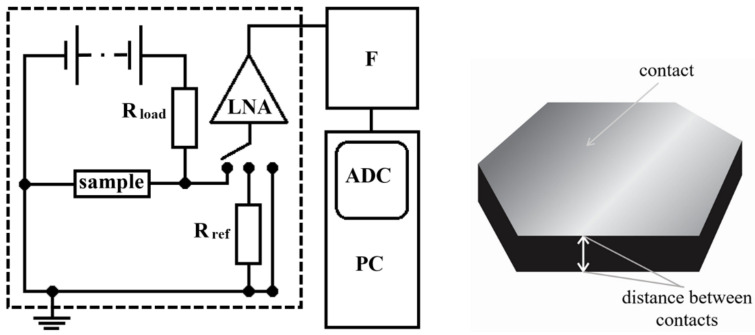
Scheme of the noise measuring system (on the left): low noise amplifier (LNA), filter system (F), analog-to-digital converter (ADC), personal computer (PC) and schematic picture of the sample (on the right).

Buehler epoThin^™ 2^ clear and very low viscosity epoxy resin was used as the polymer matrix [44]. One of the main challenges was to combine two different substances as homogeneously as possible into a composite. First, the epoxy resin was degassed overnight at 323 K at a pressure of less than 0.05 bar. Next, the OLC powder was separated into different sizes (40, 100 and 250 nm) by centrifugation, dispersed in isopropanol and then subjected to two hours of sonication. The OLC particles were distributed as uniformly as possible in isopropanol (optically homogeneous liquid) and the suspension was added to the degassed epoxy. The resulting blend was ultrasonically treated and then a hardener was added by gentle hand stirring. The composite mixture was next poured in liquid form into small molds and hardened at room temperature. It was then cured for 6 hours at 353 K [45]. The final composites were sanded, and the contacts were applied with silver paste. The samples were of irregular shapes–schematic view is presented in Figure 2. Area of the contact of different samples varied from 13 mm^2^ to 29 mm^2^, while thickness of different samples was in the range from 1.3 ± 0.01 mm to 2.2 ± 0.01 mm. The thickness of samples was measured with the caliper Fixpoint WS-SL-150 (Wentronic, Braunschweig, Germany). In this way, composites with different sizes of OLC fillers (40, 100 and 250 nm in diameter) and different weight fractions for each size of OLC in the resin (2%, 5%, 7%, 10%, 15%) were manufactured for the study. The percolation threshold of these composites was estimated to be close to 2% of the resin weight [45].

The surface of the composites was observed by scanning electron microscopy (SEM) using a JEOL JSM 6460 LV electron microscope (JEOL Ltd, Tokyo, Japan), using the detector for secondary electrons.

### 2.2. Low-Frequency Noise Measurement Technique

In this work, the resistance and low-frequency electrical noise characteristics were measured at room temperature and in a temperature range from 78 to 380 K. The voltage–resistance dependencies at room temperature were measured by a B1500A device from Keysight Technologies (Santa Rosa, CA, USA) and their variations with temperature at constant voltage were measured along with the noise. The noise (voltage fluctuation) was measured in the frequency range of 10 Hz to 20 kHz. The noise measurements were carried out in a special shielded laboratory (Faraday cage), thus avoiding parasitic electromagnetic radiation. A simplified noise measurement scheme is shown in Figure 2, the main parts of which are the Low Noise Amplifier (LNA), the Filter system (F) and the Analog-to-Digital Converter (ADC (NI TM PCI 6115)), provided from National Instruments, (Austin, TX, USA). The noise signal was recorded and processed by a personal computer (PC).

For temperature measurements, the sample under investigation was placed in a massive thermally insulated chamber. The chamber was cooled by pouring liquid nitrogen on it and heated using a heating coil built into the chamber. The temperature was measured with a thermistor placed inside the chamber.

The noise voltage spectral density was evaluated by comparison with the thermal fluctuations of the reference resistor, $R_{\mathrm{ref}}$, described by the Nyquist theorem and used as a reference. The load resistance, $R_{\mathrm{load}}$, was selected according to the resistance of the samples studied.

If the sample resistance was small (<60 kΩ), the load resistance was chosen at least 10 times higher, and the voltage fluctuations were measured at constant current mode. The spectrum of the measured signal was obtained by the fast Fourier transform (Cooley–Tukey) algorithm, and the spectral density was calculated as in Equation (1):

(1) $$S_{U}=\frac{\bar{U^{2}}-\bar{U_{s}^{2}}}{\bar{U_{\mathrm{ref}}^{2}}-\bar{U_{s}^{2}}}4k_{B}{\mathrm{TR}}_{\mathrm{ref}},$$

where $\bar{U^{2}},\;\bar{U_{\mathrm{ref}}^{2}}$ and $\bar{U_{s}^{2}}$ are dispersions of the voltage fluctuations, respectively, of the sample under study (including the noise of the measuring system), of the reference resistor (also including the noise of the measuring system), and of the measuring system in a narrow frequency band (Δf=0.1f (here f is the central frequency of the Δf)), T is the absolute temperature of the reference resistor, i.e., 290 K for all measurements, $R_{\mathrm{ref}}$ is the resistance of the reference resistor, which in these measurements was 9.7 kΩ and $k_{B}$ is Boltzmann’s constant.

Materials with low concentration of conductive fillers are close to the percolation threshold and thus have the highest resistance. Therefore, such samples were investigated using the load resistance that is at least 10 times smaller than the sample resistance (the same load resistor was used as reference). The current fluctuations were measured and recalculated to the voltage noise spectral density according to Equation (2):

(2) $$S_{U\;}=4k_{B}{\mathrm{TR}}_{\mathrm{load}}\frac{\bar{U^{2}}-\bar{U_{\mathrm{load}}^{2}}}{\bar{U_{\mathrm{load}}^{2}}}\left(\frac{R_{\mathrm{load}}+R}{R_{\mathrm{load}}}\right),$$

where $\bar{U_{\mathrm{load}}^{2}}$ is the dispersion of the voltage fluctuations of the load resistor, $R_{\mathrm{load}}$ is the resistance of the load resistor at room temperature (290 K) and R is the resistance of the sample studied at the measurement temperature.

## 3. Results

### 3.1. Electrical Characteristics

**Figure 3:**
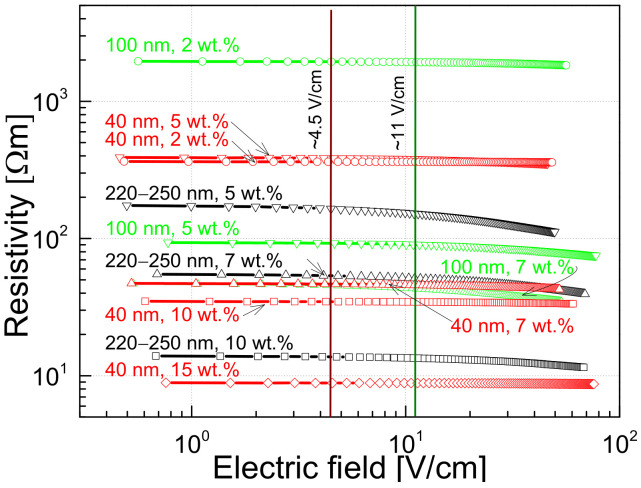
Dependences of the sample resistivity on the applied electric field at 290 K.

**Figure 4:**
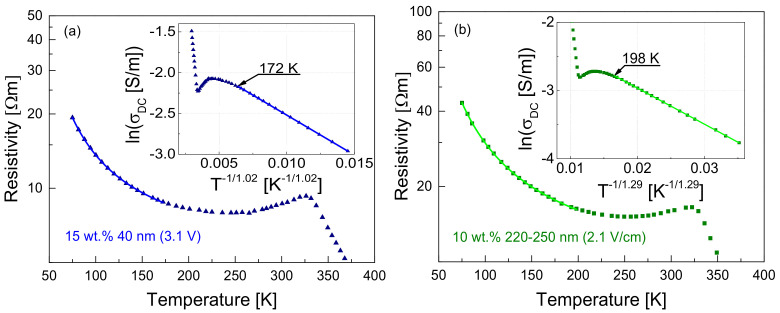
Changes of sample resistivity as a function of temperature for composites filled with 15 wt.% of OLC particles of size 40 nm (**a**), and with 10 wt.% of OLC particles of size 220–250 nm (**b**). Dots represent experimental results and solid lines represent fits for tunneling (Equation (3)). Insets represent fitting for variable-range hopping (Equation (4)). The indicated electrical field values are the values applied to the sample at room temperature (290 K).

**Figure 5:**
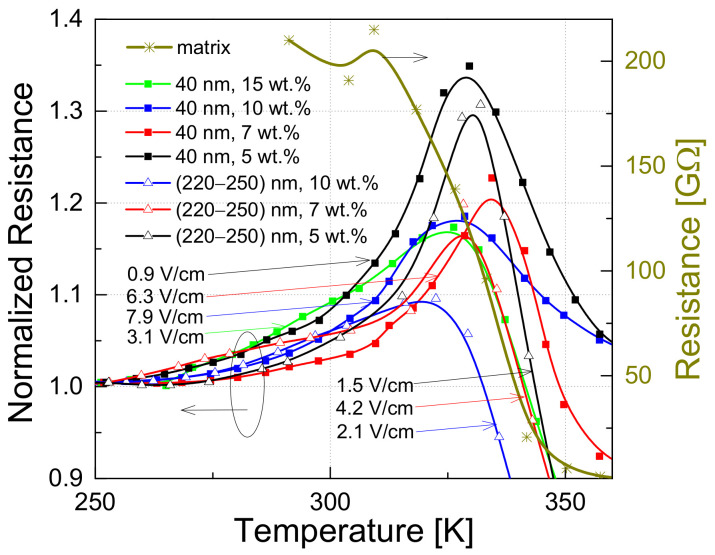
Dependence of the normalized resistance on temperature of composites and the polymer matrix, measured at different voltages depending on the sample resistance. The electrical field values applied to the sample at room temperature are indicated.

Resistivity dependencies on voltage and temperature of composites with OLC particles of different sizes and different concentration were measured. By analyzing the resistance characteristics of the investigated materials, one can note different dependencies of the measured parameters, some of them being specific to these composites only, whereas others are typical of the most polymer composites filled with carbon nanoparticles. It has been observed that the concentration of carbon fillers and their size influence the conductivity of the materials studied (Figure 3, Figure 4 and Figure 5). It has indeed also shown been in a previous work on electrical percolation in OLC/epoxy systems that the percolation threshold is the lowest in composites with large OLC particles [45].

In addition, it is clear that a higher concentration of conductive fillers decreases the resistivity of the material. As for the most composites, the investigated materials have a constant resistivity at low voltage, which starts to decrease when the voltage increases (Figure 3). However, the point at which the resistivity begins to decrease depends more on the size of the OLC particles than on the filler concentration. The resistivity begins to decrease at the lowest electric field (around 4.5 V/cm) for composites with the largest OLC particles (220–250 nm). For materials with 100 nm OLCs, this point is shifted to 11 V/cm, while for materials with 40 nm OLCs it has not been observed up to 80 V/cm (Figure 3).

The temperature dependencies of the resistance are similar for all investigated composites: the resistivity decreases with increasing temperatures of up to 220–260 K (Figure 4) and increases with temperatures in the region of approximately 250–325 K (depending on filler size and concentration), while at higher temperatures the resistivity decreases due to the onset of matrix conductivity [46]. Figure 5 highlights the temperature region where matrix expansion increases the sample resistance. Since this is a large relative—not absolute—increase in resistance, the resistance of each sample has been normalized to its value at 250 K, i.e., where the increase in resistance starts. Therefore, the normalized resistance of all samples coincides at 250 K and is equal to one. The matrix resistance is constant up to approximately 300 K (Figure 5). Given that the molecular structure of the epoxy resin does not change in the temperature range 250–300 K [46,47], its physical expansion is thus responsible for the phenomenon observed. This leads to the separation of the conductive OLC particles from each other, which increases the resistance of the composite. The impact of matrix expansion is more obvious for composites with smaller particles and lower concentration, i.e., in materials closer to the percolation threshold, the peak of resistance is relatively higher.

In composite materials where the filler concentration exceeds the percolation threshold, but it is not very high, the space between adjacent conductive particles can be seen as the contact barrier, which can be quite large. The electrical conduction in such a structure, depending on the temperature, can be the result of tunneling and variable-range hopping [40,48,49]. Therefore, the resistivity-temperature characteristics of all investigated materials at low temperature, i.e., under which there is no effect of matrix expansion, have been approximated by the equation for charge carrier tunneling [48] and by the Mott’s equation for variable-range hopping processes [50]. As the investigated materials have similar characteristics at low temperature, Figure 4 represents the fitting results for tunneling and for variable-range hopping for two samples with 10 wt.% of OLC particles of the size 220–250 nm. and 15 wt.% of OLC particles of size 40 nm.

**Figure 6:**
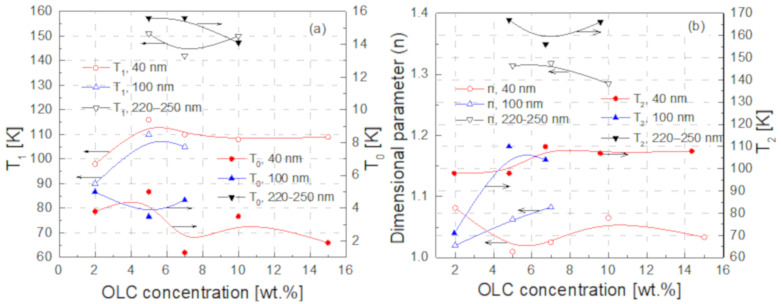
Parameters for fitting the experimental data in Figure 3, as a function of filler concentration for different sizes of OLC: T_0_ and T_1_ from the fit by Equation (3) (**a**), and dimensional parameter n and parameter T_2_ from the fit by Equation (4) (**b**).

The fluctuation-induced tunneling can be expected at low temperature in composite materials [48,49]. This process is described by Equation (3):

(3) $$\rho =\rho_{0}\exp\left(\frac{T_{1}}{T+T_{0}}\right),$$

where T is the sample temperature, T_0_ is the temperature below which the DC tunneling is independent of temperature and T_1_ reflects the energy required to transfer charge carriers between the OLC particles. Equation (3) fitted the experimental results at low temperatures well (Figure 4). This suggest that, in the range 75–180 K for composites with 40 nm and 100 nm OLC particles, and in the range 75–225 K for composites with 220–250 nm particles, tunneling between OLC particles or their clusters occurs. However, the parameter T_0_ in all investigated materials was below the temperatures studied (Figure 6a). Thus, in the investigated temperature range, the charge carriers tunneling is caused by thermal activation. The values of the parameter T_1_ are also presented in Figure 6a. Both fitting parameters T_0_ and T_1_ weakly depend on the OLC particle concentration.

The resistance characteristics of the materials investigated at low temperature were also well fitted by the Mott’s equation for variable-range hopping [50]:

(4) $$\rho =\rho_{0}\exp{\left(\frac{T_{2}}{T}\right)}^{\frac{1}{n}},$$

where n=1+d and d is the dimensionality of the conductive structures, while $T_{2}$ reflects the energy required for carrier jump to the nearest-neighboring state. Therefore, the variable-range hopping is also present in the investigated materials. The results of the fits by the Mott’s formula are presented in insets in Figure 4, enabling us to find the dimensionality of the transport as seen by the charge carriers (Figure 6b). There is no clear dimensional dependence of the particles concentration, but it is much more important for larger particles. The dimensional parameter *n* equal to 1.0–1.1 for 40 nm OLC particles shows that the quasi-one-dimensional electrical transport dominates, while the *n* value around 1.3 for 220–250 nm particles suggests the beginning of a transformation towards two-dimensional transport. The dimensionality of the electrical transport in the investigated composites is in good agreement with that observed in OLC powders [7]. Therefore, the hopping of electrons occurs mainly inside the OLC clusters between neighboring carbon atoms. The parameter $T_{2}$ also takes higher values for larger OLC particles, which indicates a higher concentration of defects in larger OLCs, and it weakly depends on their concentration (Figure 6b).

**Figure 7:**
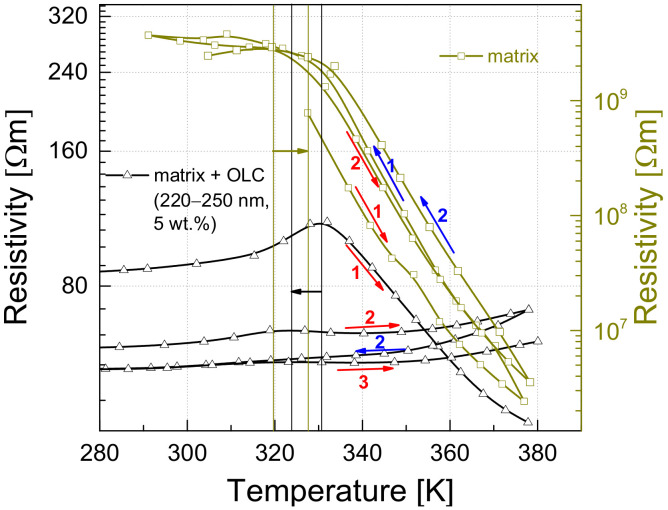
Dependence of the resistivity on the temperature for the composite with 5 wt.% of OLCs of size 220–250 nm and the epoxy resin with repeated cooling and heating cycles: red arrows indicate heating, blue arrows indicate cooling and the numbers near them indicate the cycle number. The vertical lines indicate the onset of noticeable changes in the structure, and the horizontal arrows indicate the direction of this onset point during the repetition of the cycles.

It has been noted that the resistance characteristics of the investigated materials vary with repeated measurement cycles, and this variation is most evident in the peak region (above 250 K). Therefore, successive measurements were carried out (Figure 7). After the first measurement (heating), the sample was taken out of the system; the second measurement was also performed during heating, while the other measurement cycles (as well as matrix measurements) were performed without interruption. The rapid expansion of the epoxy resin occurs above the glass transition temperature, which can be about 250–410 K for pure epoxy resin [47]. The resistivity of all measured composites during the first heating cycle starts to increase from approximately 250 K and reaches a maximum at around 325 K (Figure 5). However, a change in the resistivity of the investigated composites has been observed during repeated heating-cooling cycles (Figure 7); when the material is cooled from a temperature above the glass transition temperature of the polymer, its peak resistivity decreases and, during the following heating cycle, the increase in resistivity is not as sharp as during the first cycle. The repetition of heating-cooling cycles up to 380 K leads to an almost unobserved increase in resistivity. This result shows that a redistribution of OLC particles and polymer chains occurs when the sample is cooled from a temperature above 250 K. It has also been observed that the temperature at which the matrix resistivity begins to decrease has moved towards a higher value, while the temperature at which the composite resistivity is at its maximum has decreased (vertical lines in Figure 7). Another characteristic observed during the repetition of the measurements is that the appearance of conductivity in the polymer matrix at a temperature above 325 K becomes insignificant: there is a small, steady increase in resistivity with increasing temperature (Figure 7). Considering that the slope of the decrease in matrix resistivity has not changed after repeated measurements and that the resistivity returns to its initial value at low temperature, it can be concluded that during repeated heating-cooling cycles, the OLC particles redistribute themselves in the matrix. Therefore, the conductivity due to the percolation network is larger and, therefore, the matrix expansion is much less significant. 

### 3.2. Noise Characteristics

**Figure 8:**
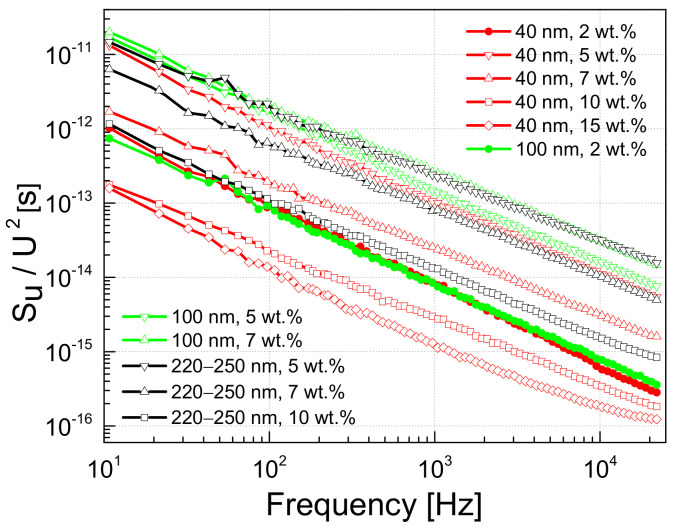
Spectra of the voltage noise for composites with different filler concentrations and different OLC particle sizes at 290 K.

The composite materials considered can be seen, in general, as disordered structures with defects in the polymer matrix as well as imperfections in the outer shells of the OLC particles [4,6]. These defects form charge carrier trapping centers with widely distributed characteristic times, and the processes of capture and release of charge carriers through these centers are well reflected in the low-frequency noise characteristics. The voltage noise spectra of the materials investigated at room temperature are 1/f ^α^–type, where α varies between 0.68 and 1.45 for different spectra (Figure 8), which is characteristic of the superposition of the processes of capture and release of charge carriers by the trapping centers with widely distributed characteristic times [37,41,49,51].

**Figure 9:**
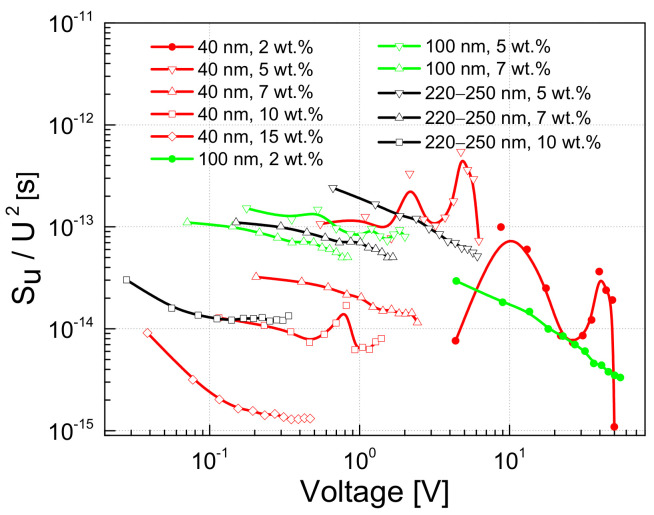
Dependence of the voltage noise spectral density on voltage for composites with different filler concentrations and different OLC particle sizes at 290 K.

In the region where the resistance does not depend on the voltage (approximately below 1 V (Figure 3)), the magnitude of the voltage noise spectral density depends on the concentration of the OLC particles; a higher concentration of conductive OLC particles results in a lower noise intensity and is almost independent of the particle size (Figure 9). However, this dependence is more significant for composites filled with the smallest OLC particles (40 nm).

The voltage noise spectral density is proportional to the particular exponent of the voltage: $S_{U}\sim U^{b}$, where 1<b<2 (Figure 9). The exponent b=2 is characteristic of resistance fluctuations due to processes of generation and recombination of charge carriers [35]. The value of *b* decreases when the voltage increases due to a greater contribution to the materials conductivity of tunneling processes across the different potential barriers.

**Figure 10:**
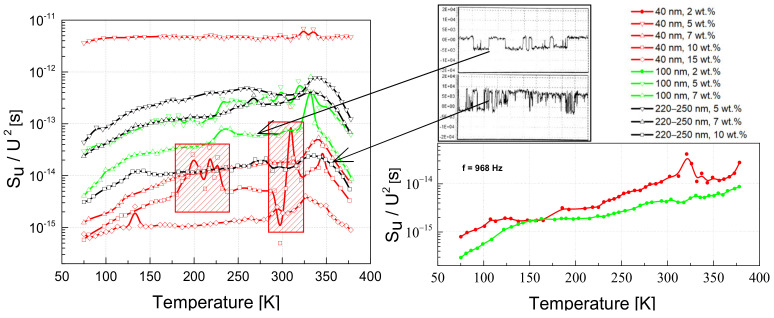
Dependence of the voltage noise spectral density on temperature for different composites with OLC particles, at a fixed frequency of 968 Hz. The shaded red rectangles denote the region where impulse noise was observed; impulse noise oscillograms are presented in the inset.

In general, the variations in voltage noise spectral density as a function of temperature do not present noticeable particularities for these composites based on OLC particles (Figure 10) compared to those, for example, based on carbon nanotubes [49]. There is a tendency for materials with larger particles to have larger voltage fluctuations, but exceptions are also present, e.g., the sample with 5 wt.% of 40 nm particles (Figure 10). The investigated materials have resistance that decreases with temperature up to about 250 K, and the corresponding noise intensity increases slowly. However, some samples in particular temperature regions showed impulse noise. This instability is more inherent to composites with smaller OLC particles. For example, the sample with 10 wt.% of 40 nm OLC particles has impulse noise from 180 to 230 K, and from 280 to 325 K (Figure 10). The second temperature region coincides with matrix expansion. All investigated samples showed a more or less significant increase in the noise intensity with temperature, where the conductivity of the matrix becomes important (about 330 K).

**Figure 11:**
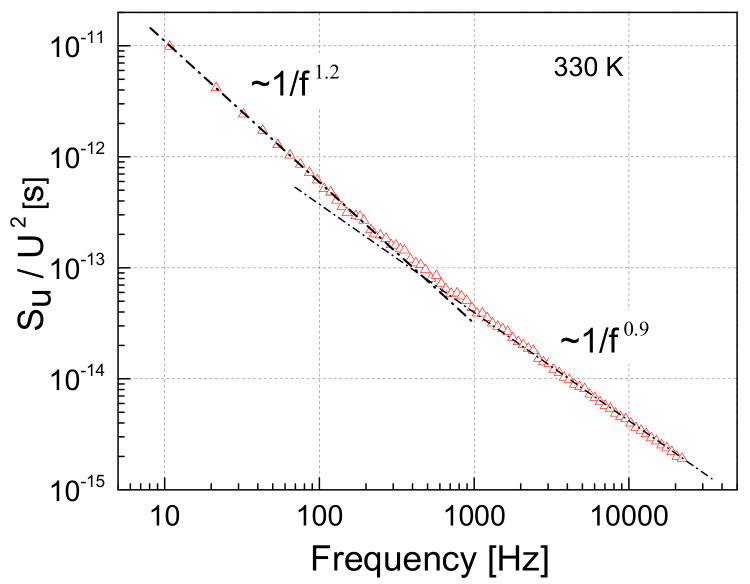
Voltage noise spectrum at 330 K, where matrix expansion starts to occur, for the sample with 7 wt.% of 40 nm OLC particles.

The character of the voltage noise spectra does not noticeably change at different temperatures: it is 1/f ^α^–type. However, while at room and lower temperature, the exponent α is constant over the investigated frequency range, it varies with frequency in the temperature region where the influence of matrix expansion is observed (from around 330 K). An example of such a spectrum is presented in Figure 11: at lower frequencies, α is 1.2, while at higher frequencies it is equal to 0.9.

**Figure 12:**
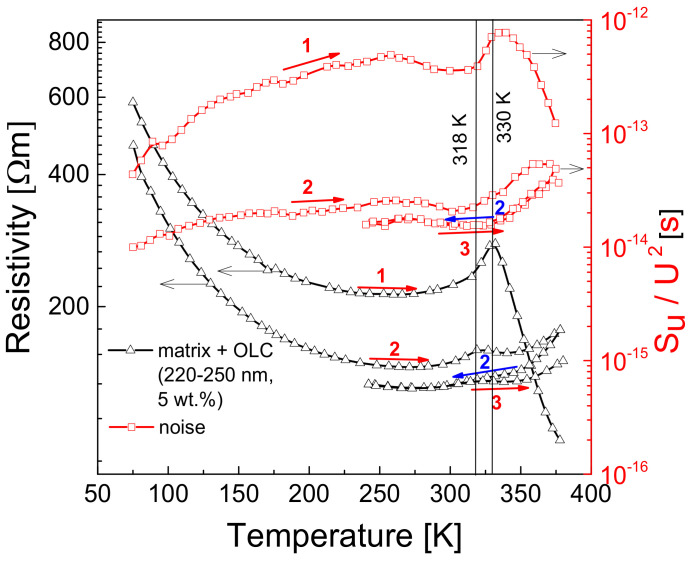
Resistivity and the voltage noise dependencies on temperature for a material with 5 wt.% of (220–250) nm OLC particles during repeated cycles of cooling (blue arrows) and heating (red arrows). The numbers indicate the cycle number.

In the previous section (Figure 7), it was shown that the resistance of the investigated materials decreases during repeated heating and cooling cycles. A similar behavior was noticed in the noise characteristics (Figure 12), using the same repetition procedure as for the resistance measurements. Similar to the resistivity dependencies after repeated measurements, there is no noise peak when the matrix expands, and the noise spectral density increases with temperature, where an initial decrease was observed (correlating with the decrease in composite resistivity due to the onset of matrix conductivity). Moreover, it can be concluded that the temperature behavior of the noise characteristics and that of the resistivity are correlated, except in the low temperature region (below 150 K), where the decrease in noise is followed by the increase in resistance. The correlation between these two quantities was found to be in good agreement with the percolation theory, as shown in Equation (5):

(5) $$\frac{S_{U}}{U^{2}}\approx R^{\frac{k}{t}},$$

where k/t can range from 0.87 to 3.2 for various random-void models [52].

At low temperatures, the noise behavior can be described with the two-level tunneling system fluctuator model [53] as shown in Equation (6):

(6) $$S_{U}=\frac{\mathrm{AT}}{R^{2}}.$$

This confirms the importance of the electron tunneling mechanism for electrical transport. However, for hopping conductivity, opposite behaviors of noises and the resistivity at low temperatures can also be explained [54].

## 4. Discussion

**Figure 13:**
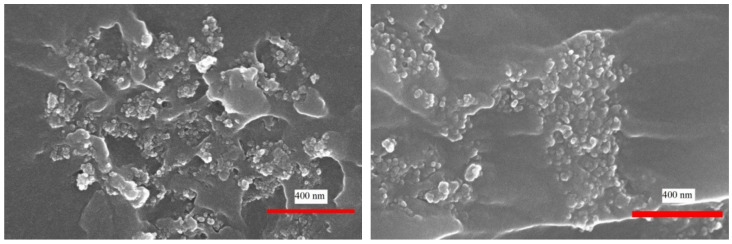
Scanning electron microscopy images of composites with 10 wt.% of OLC particles of size 40 nm (left) and 220–250 nm (right).

A composite material with carbon nanofillers can be understood as a dielectric matrix containing conductive particles that can form a percolation network. Such a material, in general, has a disordered structure. There are contact barriers between the carbon particles, and, therefore, the charge carriers must tunnel or overflow the barriers to ensure electrical conductivity. These mechanisms of charge carrier transfer can be induced by temperature and fluctuations of free carriers [55]. Therefore, the electrical characteristics of composite materials may depend differently on temperature, voltage and other operation conditions. Moreover, spheres of onion-like carbon structures are not perfect: the external shells are incomplete and/or imperfectly organized. Otherwise, if the spheres were perfect, they would be dielectric like fullerenes [4,6]. These defects form centers for charge capture and release, which enhance the processes of charge carrier generation and recombination, leading to a fluctuation in the number of free charge carriers. OLC particles can connect to each other through incomplete spheres (defective graphene-like sheets), producing a direct contact. In this case, there are no polymer barrier between the particles. Assuming that this direct contact connection is more frequent for larger OLCs (and indeed, larger clusters are observed in composites with bigger OLCs (Figure 13)), this would explain why composites with smaller particles need a higher voltage to reach the point where the resistance starts to decrease, due to tunneling through the barriers (Figure 3). The barrier-type connection is more common in composites with small particles than in materials with larger particles.

The particle size has an insignificant impact on the low-frequency voltage noise characteristics (Figure 8 and Figure 9). However, it has been observed that composites with small particles (40 nm) are more sensitive to any change in operating conditions. Firstly, the resistivity and voltage noise density increase due to matrix expansion is larger in the 250–320 K range (Figure 5). Secondly, the impact of matrix expansion on the resistivity characteristic (the resistivity begins to increase) starts at a lower temperature. Third, the voltage noise spectral density has greater instabilities as a function of voltage and temperature (Figure 9 and Figure 10). These results lead to the conclusion that small OLC particles are less connected by direct contact, which makes it easier to move them away from each other as the matrix expands. This is consistent with the fact that the percolation threshold is lower in composites filled with larger nanoparticles [45]. For the same OLC concentration, the conductivity of the percolation network is higher for composites with larger OLCs, while the impact of the properties of the epoxy matrix is less important. Yanashima et al [56] observed that the interaction between the epoxy resin and fillers is significantly increased when the size of the filler decreases. On the other hand, charge carrier trapping centers at the incomplete external OLC shells increase the fluctuation of the number of carriers in the sample by enhancing the carrier generation and recombination processes. The activity of these centers depends on the operation conditions (voltage and temperature) [36,37].

A higher concentration of conductive OLC particles obviously leads to a higher conductivity of the material. But it also leads to a lower voltage noise intensity, which can be explained by the larger number of fluctuation origins, which partially cancel each other out, and, therefore, the overall noise intensity is lower (and 1/f noise spectra are usually observed [38]). The variable slope of the voltage noise spectra over the investigated frequency range at temperatures close to that where the resistivity peak (due to the matrix expansion) is observed, shows that additional charge carrier capture and release centers are activated.

The temperature characteristics of the resistivity of the investigated composites are well fitted by Equations (3) and (4) (Figure 4), indicating that tunneling between the OLC particles or their clusters and variable-range hopping of the charge carriers inside the OLC clusters contribute mainly to the electrical conduction in the investigated materials. The 1/f type voltage fluctuation shows that the number of free charge carriers in the material fluctuates due to generation and recombination processes. The fitting results show that the tunneling of charge carriers through the potential barriers in the studied temperature range (above 75 K) is caused by thermal activation, since the parameter *T*_0_ in Equation (3) was below the temperatures investigated here.

The noise characteristics confirm the presence of more than one process of charge carrier transfer: thermally activated tunneling, variable-range hopping and charge carrier generation and recombination contribute to the conductivity of the investigated materials. Quasi-one-dimensional electron hopping inside OLC clusters dominates the electrical transport in these composites.

The redistribution effect of OLC particles inside epoxy resin during heating/cooling measurements up to 380 K is followed by simultaneously decrease of the resistivity and the voltage noise, in good agreement with the percolation theory.

## 5. Conclusions

The electrical resistivity and low-frequency voltage noise characteristics of composite materials based on an epoxy matrix filled with onion-like carbon nanoparticles were investigated. The predominant processes of charge carrier transfer, until the matrix conductivity takes place, are by temperature-activated tunneling between the conductive OLC particles and by variable-range hopping inside the particles or their clusters, as well as by charge carrier generation and recombination through trapping centers within the nanoparticles. Quasi one-dimensional electrical transport is observed in composites with the smallest particles (40 nm), and dimensionality of electrical transport is slightly larger in the composites with larger OLC particles (220–250 nm) due to the more defective structure of larger OLCs.

The characteristics of the electrical conductivity depend on the size of the OLC particles and their concentration. The observed 1/f–type electrical fluctuations are a result of superposition of generation and recombination processes that cause fluctuation of number of free carriers in the material. 1/f–type noise intensity is lower in composites with a larger concentration of OLC particles as larger number of noise origins partially offsets the contribution of each other. Characteristics of the materials with smaller particles are more sensitive to the physical changes caused by temperature changes in the polymer matrix. Smaller OLC particles are less connected by direct contact (there is contact barrier), which makes it easier to move them away from each other or redistribute as the matrix expands.

## Data Availability

The data presented in this study are available on request from the corresponding authors.
